# Healthcare Providers’ Perceptions and Self-Reported Fall Prevention Practices: Findings from a Large New York Health System

**DOI:** 10.3389/fpubh.2015.00017

**Published:** 2015-04-27

**Authors:** Matthew Lee Smith, Judy A. Stevens, Heidi Ehrenreich, Ashley D. Wilson, Richard J. Schuster, Colleen O’Brien Cherry, Marcia G. Ory

**Affiliations:** ^1^Department of Health Promotion and Behavior, College of Public Health, The University of Georgia, Athens, GA, USA; ^2^National Center for Injury Prevention and Control, Centers for Disease Control and Prevention, Atlanta, GA, USA; ^3^Department of Health Promotion and Community Health Sciences, Texas A&M Health Science Center School of Public Health, College Station, TX, USA; ^4^Center for Global Health, College of Public Health, The University of Georgia, Athens, GA, USA

**Keywords:** clinical practice, fall prevention, fall screening, intervention science

## Abstract

Among older adults, falls are the leading cause of injury-related deaths and emergency department visits, and the incidence of falls in the United States is rising as the number of older Americans increases. Research has shown that falls can be reduced by modifying fall-risk factors using multifactorial interventions implemented in clinical settings. However, the literature indicates that many providers feel that they do not know how to conduct fall-risk assessments or do not have adequate knowledge about fall prevention. To help healthcare providers incorporate older adult fall prevention (i.e., falls risk assessment and treatment) into their clinical practice, the Centers for Disease Control and Prevention’s (CDC) Injury Center has developed the Stopping Elderly Accidents, Deaths, and Injuries (STEADI) tool kit. This study was conducted to identify the practice characteristics and providers’ beliefs, knowledge, and fall-related activities before they received training on how to use the STEADI tool kit. Data were collected as part of a larger State Fall Prevention Project funded by CDC’s Injury Center. Completed questionnaires were returned by 38 medical providers from 11 healthcare practices within a large New York health system. Healthcare providers ranked falls as the lowest priority of five conditions, after diabetes, cardiovascular disease, mental health, and musculoskeletal conditions. Less than 40% of the providers asked most or all of their older patients if they had fallen during the past 12 months. Less than a quarter referred their older patients to physical therapists for balance or gait training, and <20% referred older patients to community-based fall prevention programs. Less than 16% reported they conducted standardized functional assessments with their older patients at least once a year. These results suggest that implementing the STEADI tool kit in clinical settings could address knowledge gaps and provide the necessary tools to help providers incorporate fall-risk assessment and treatment into clinical practice.

## Introduction

Falls are the leading cause of death and emergency department visits for injury among older adults (1), and the direct medical costs for these injuries are estimated to be more than $30 billion dollars annually (2). Falls are caused by a number of risk factors usually classified as either intrinsic (e.g., age, sex, chronic diseases, medication side effects, gait and/or balance problems, muscle weakness) or extrinsic (e.g., environmental factors such as uneven surfaces, poor lighting, and lack of railings and/or grab bars) (3–7). It is expected that the incidence of falls and associated injuries will continue to rise as the nation’s population of older adults increases. However, fall risk can be reduced through multifactorial interventions that are implemented in clinical settings (8, 9).

The American and British Geriatrics Societies (AGS/BGS) have published a clinical practice guideline to reduce falls (10). However, primary care physicians have been slow to put the AGS/BGS guideline into clinical practice because many feel that they do not know how to conduct fall-risk assessments or do not have adequate knowledge about fall prevention (11, 12). To help healthcare providers incorporate older adult fall prevention into their clinical practice, experts at Centers for Disease Control and Prevention’s (CDC) Injury Center developed the Stopping Elderly Accidents, Deaths, and Injuries (STEADI) tool kit. The tool kit is based on the AGS/BGS clinical practice guideline (10), applies concepts from Wagner’s Chronic Care Model (CCM) (13) to fall risk, and includes input from healthcare providers (14). It contains basic information about falls, standardized gait and balance assessment tests, case studies, and conversation starters. In addition, there are educational handouts about fall prevention specifically designed for older patients and their friends and family. The contents of the STEADI tool kit and supplemental resources are available online (15).

Data for this study were collected as part of a larger 5-year project begun in 2011. This project funded three state health departments (Colorado, New York, Oregon) to integrate clinical and evidence-based community fall prevention programs in selected communities. This study describes the beliefs, knowledge, and fall-related activities of 38 healthcare providers from 11 healthcare practices within a large New York health system, prior to receiving training about implementing the STEADI tool kit. This community case study describes the current attributes, perceptions, and self-reported practices of healthcare providers. The results underscore the need to enhance providers’ knowledge about fall prevention and for clinical resources to support falls screening, assessment, and treatment.

## Materials and Methods

At part of a cooperative agreement with the CDC, the STEADI evaluation and implementation teams (led by the Texas A&M Health Science Center and The University of Georgia, respectively) developed a provider training based on academic detailing called the Clinical Engagement and Education (CEE) session (16). These teams trained the state grantees to conduct CEE sessions and developed, tested, and refined the evaluation materials and processes.

The purpose of the CEE session was to help clinicians find innovative ways to incorporate STEADI into their clinical practice (16). The 1-h session was led by a physician fall prevention “Champion” who had been identified and trained by the state grantee, and was open to all clinicians and office personnel in the practice. These interactive sessions were designed to bring healthcare providers and office staff together to discuss the burden of older adult falls and to foster collective decisions about fall prevention activities that they could implement during clinical visits with older adult patients (16).

### Data collection

Clinical Engagement and Education session data about the characteristics of the practice, provider characteristics, and provider beliefs, knowledge, and fall-related activities were collected from two sources. First, office personnel completed a registration form after the practice agreed to participate in the CEE session. This form provided general information about the healthcare group (e.g., number of years the practice has been in business, the number of employees, size of the patient base).

Second, each CEE session participant was asked to complete a two-page questionnaire at the beginning of the CEE Session. The 35-item questionnaire took approximately 15 min to complete and asked for the participant’s characteristics (i.e., job title, gender), opinions about fall-risk factors, practice priorities, and activities conducted during clinical visits with older patients. Responses consisted of Likert scales and closed-response formats. Institutional Review Board approval was obtained from Texas A&M University to conduct descriptive analyses using de-identified data.

### Measures

Providers were asked to rate each of five health conditions from 1 (low) to 10 (high) in response to the question, “When thinking about your older patients, please rate the level of priority given to conditions in your practice.” Then, given a list of eight fall-risk factors, providers were asked to, “Rate the extent to which you believe the following items are fall-risk factors for your older patients.” Each factor was rated from 1 (low) to 10 (high). Finally, they were asked, “In the past month, approximately what percent of your older patients have you referred to attend community fall prevention programs?”

Given a list of 10 intervention activities, providers were asked to report the proportion of older patients who received specific fall interventions at least once a year. Examples of intervention activities included discussing prescription medications, discussing mobility aids, assessing visual acuity, and performing standardized physical functioning assessments. Responses were measured using five-point Likert scales but, based on the frequency distribution, these were collapsed into three categories: none, a few or some, and most or all.

Other items asked of providers, but not presented in tables, included the average amount of time (in minutes) they spent with an older patient during a typical visit and the average amount of time (in minutes) they spent assessing fall risk during a typical visit with an older patient. Providers were also asked their level of agreement with statements including, “My older patients are reluctant to tell me they have fallen;” “It is important to perform a standardized fall-risk assessment with older adults;” “Gait and balance tests are easy to perform;” and “I have adequate time during a clinical visit to assess fall risk among my older patients.” Responses were measured using four-point Likert scales but, based on the frequency distribution, these were collapsed into two categories: agree and disagree.

### Statistical analyses

Given the limited number of participants in this study, data are described but no tests for statistical significance were performed. Some data are presented in tabular form and others are described in the text.

## Results

### Practice characteristics

Between September 2012 and June 2013, 11 New York based practices within United Healthcare, a managed healthcare company, hosted 11 CEE sessions. These practices had existed for an average of 20 years (range: 5–30 years). Each practice served an average of 6,365 patients (range: 320–12,000 patients) and, on average, 43% of these patients (range: 20–70%) were aged 65 years or older. Each practice employed an average of 15 medical personnel (range: 4–30 employees) that included between one and six physicians.

### Provider characteristics

Forty-nine persons attended the CEE sessions. For this study, office personnel (*n* = 5) and those with missing socio-demographic data (*n* = 6) were excluded. Therefore, data are presented for 38 medical providers. Of these, 34% were nurses, 26% physicians, 18% nurse practitioners, 8% physician assistants, 8% medical assistants, and 3% specialty care providers. The median age was 38 years (range: 23–69 years), and 84% were female.

### Provider beliefs and knowledge

Table 1 shows the participants’ level of priority given to specific health conditions and beliefs about fall-risk factors among older adults. Of the five health conditions, diabetes received the highest average score (8.4) while falls received the lowest (7.1). Of eight fall-risk factors, a history of falling received the highest average score (8.1). Postural hypotension received the lowest average score (6.1).

**Table 1:** **Healthcare providers’ priorities and beliefs about the importance of issues facing older adult patients**.

	*N*	Median	Mean	SD	Range	
					Minimum	Maximum
**Priority given to health conditions^a^**
Diabetes	37	9	8.35	1.69	3	10
Cardiovascular disease, including stroke	37	8	8.08	1.99	3	10
Mental health, including depression	34	8	7.44	2.22	3	10
Musculoskeletal conditions	37	8	7.35	1.93	3	10
Falls	37	7	7.05	2.15	3	10
**Beliefs about fall-risk factors for older patients^a^**
History of falling	38	9	8.11	2.35	3	10
Balance issues	38	9	7.95	2.27	3	10
Gait issues	37	9	7.68	2.46	2	10
Environmental issues within the home	38	8	7.16	2.09	3	10
Medication issues	38	8	7.13	2.47	2	10
Neurological issues	38	7	6.68	2.35	2	10
Vision issues	35	7	6.66	2.35	2	10
Postural hypotension	36	6	6.11	2.51	2	10

*^a^All items measured on a scale from 1 = low to 10 = high*.

### Provider fall-related activities

Providers reported that a typical office visit with an older patient lasted on average 20.7 (±9.9) minutes (range: 0–60 min). The time spent assessing fall-risk factors averaged 3.8 (±2.5) minutes (range: 0–10 min). Approximately, 66% of respondents agreed or strongly agreed with the statement, “I have adequate time during a clinical visit to assess fall risk among my older patients.”

Table 2 shows the proportion of respondents who delivered specific fall interventions to their older patients at least once a year. Over 81% of providers discussed details about prescribed medications with most or all of their older patients. About 47% conducted a cognitive screening with most or all of their older patients, and 37% asked most or all of their older patients about falls during the past 12 months.

**Table 2:** **Proportion of older patients for whom activities are performed at least once a year**.

	*N*	None (%)	A few/some (%)	Most/all (%)
Discuss details about their prescribed medications (e.g., number, type, dose, side effects)	37	0.0	18.9	81.1
Conduct a cognitive screening	34	11.8	41.2	47.1
Discuss their use of mobility aids	37	5.4	56.8	37.8
Collect fall history over the past 12 months	38	18.4	44.7	36.8
Educate about their specific fall-risk factors	36	16.7	52.8	30.6
Follow-up with patients who are at risk for falling within 30 days of their clinical visit	36	16.7	55.6	27.8
Assess their visual acuity	35	5.7	77.1	17.1
Conduct the *Timed Up and Go* test	32	53.1	31.3	15.6
Conduct the *30-s Chair Stand* test	34	58.8	32.4	8.8
Conduct the *4-Stage Balance* test	35	65.7	25.7	8.6

All providers reported that it was important to perform a standardized fall-risk assessment and said gait and balance tests were easy to perform. Just over one-third of the providers routinely asked their older patients if they had fallen in the past year. Yet, about 61% of providers agreed or strongly agreed with the statement, “My older patients are reluctant to tell me they have fallen.” As shown in Table 2, fewer than 16% reported that they conducted either the *Timed Up and Go* test, *30-s Chair Stand*, or *4-Stage Balance Test* with most or all of their older patients at least once a year.

Thirty-one providers reported that they referred on average 20% (±18.5%) of their older adult patients to community fall prevention programs (range: 10–100%). Similarly, 32 providers reported that they referred on average 22% (±18.8%) of their older patients to physical therapy for gait and/or balance retraining (range: 10–100%).

## Discussion

This study examined the beliefs, knowledge, and fall-related activities conducted among 38 healthcare providers. These data, collected at the beginning of the CEE session, showed that the providers considered all five specified health conditions were high priority. However, falls were considered a lower priority than chronic conditions such as diabetes and cardiovascular disease.

Despite clinical guidelines (10), few providers routinely asked their older patients if they had fallen in the past year. This is especially troubling since providers reported that their patients were reluctant to tell them that they had fallen. These data further indicated that few providers actually conducted standardized tests to assess gait and balance, although these tests are seen as both important and easy to perform. The low assessment rate by providers was partially counterbalanced by patient referrals to physical therapy to address gait or balance problems.

Prior research suggests that primary care providers feel that they do not know how to conduct fall-risk assessments and this study found that providers were not conducting multifactorial risk assessments on every patient (11, 12, 17). These are missed opportunities for prevention that are likely to result in higher fall rates. An important next step is to make fall prevention a routine part of clinical care. This requires educating providers about how to conduct fall-risk assessments and providing them with the necessary tools to streamline the process. Promising approaches include educating providers about the STEADI tool kit as well as providing them with additional resources such as online provider education and clinical decision support modules that are integrated into the provider’s electronic health records (EHR) system.

## Limitations

A limitation of this study is that provider data were available for only 38 healthcare providers from one healthcare organization in one state, so findings must be considered preliminary. While study data were obtained from a diverse set of healthcare providers, the small number of respondents made it impossible to examine how the knowledge, beliefs, and activities differed by provider or practice type. Further investigation is warranted to assess such differences. Additionally, these data were collected pre-intervention, before the healthcare providers were introduced to the tool kit. Because insufficient follow-up data were collected post-intervention, changes in healthcare providers’ beliefs and behaviors could not be assessed. Further investigation is warranted to examine the impact of the STEADI tool kit on healthcare providers’ perceptions and clinical practice.

The approach used for recruiting healthcare practices to receive training in using the STEADI tool kit may have limited participation; it may have selected those participants who were especially interested in fall prevention. This would suggest that the frequency of fall-risk assessments (e.g., collecting fall history, conducting standardized gait, and balance tests) actually might be lower among healthcare providers.

Another limitation was associated with hosting CEE sessions in healthcare practices. State health department grantees were required to first identify and then train a highly motivated provider Champion. This was a difficult and labor-intensive process because the grantees had to first establish new partnerships with healthcare provider groups. Future efforts will include developing an online training for STEADI with continuing education (CE) credits, and creating a software module to integrate STEADI’s fall prevention processes into EHR.

Lastly, the study relied on self-reported estimates of fall prevention activities that could not be confirmed by objective measures such as medical chart reviews. These estimates may not accurately reflect the true frequencies of these activities in primary care settings.

## Conclusion

This study found that most healthcare providers did not consider falls as high a priority as other chronic conditions among older patients, and did not routinely assess and address these patients’ fall-risk factors. The STEADI tool kit may be a valuable resource to help providers incorporate fall-risk assessment, treatment, and/or referral into clinical practice. However, providers must first be convinced that falls are a priority issue among their older patients, and devote as much, or more, time to assessing falls risks and educating patients about appropriate programs to reduce fall risks. Future studies will focus on educating providers about the STEADI tool kit, their adoption of STEADI, and STEADI’s impact on fall-risk screening, assessment, and treatment.

## Conflict of Interest Statement

The authors declare that the research was conducted in the absence of any commercial or financial relationships that could be construed as a potential conflict of interest.

This paper is included in the Research Topic, “Evidence-Based Programming for Older Adults.” This Research Topic received partial funding from multiple government and private organizations/agencies; however, the views, findings, and conclusions in these articles are those of the authors and do not necessarily represent the official position of these organizations/agencies. All papers published in the Research Topic received peer review from members of the Frontiers in Public Health (Public Health Education and Promotion section) panel of Review Editors. Because this Research Topic represents work closely associated with a nationwide evidence-based movement in the US, many of the authors and/or Review Editors may have worked together previously in some fashion. Review Editors were purposively selected based on their expertise with evaluation and/or evidence-based programming for older adults. Review Editors were independent of named authors on any given article published in this volume.
